# Maternal and paternal effects on offspring internalizing problems: Results from genetic and family‐based analyses

**DOI:** 10.1002/ajmg.b.32784

**Published:** 2020-05-01

**Authors:** Eshim S. Jami, Espen Moen Eilertsen, Anke R. Hammerschlag, Zhen Qiao, David M. Evans, Eivind Ystrøm, Meike Bartels, Christel M. Middeldorp

**Affiliations:** ^1^ Department of Biological Psychology Vrije Universiteit Amsterdam Amsterdam The Netherlands; ^2^ Amsterdam Public Health Research Institute Amsterdam The Netherlands; ^3^ Department of Mental Disorders Norwegian Institute of Public Health Oslo Norway; ^4^ Child Health Research Centre University of Queensland Brisbane Australia; ^5^ The University of Queensland Diamantina Institute The University of Queensland Brisbane Australia; ^6^ Medical Research Council Integrative Epidemiology Unit at the University of Bristol Bristol UK; ^7^ Population Health Sciences, Bristol Medical School University of Bristol Bristol UK; ^8^ PROMENTA Research Center, Department of Psychology University of Oslo Oslo Norway; ^9^ School of Pharmacy University of Oslo Oslo Norway; ^10^ Child and Youth Mental Health Service Children's Health Queensland Hospital and Health Service Brisbane Australia

**Keywords:** anxiety, depression, genetic nurture, M‐GCTA, MoBa

## Abstract

It is unclear to what extent parental influences on the development of internalizing problems in offspring are explained by indirect genetic effects, reflected in the environment provided by the parent, in addition to the genes transmitted from parent to child. In this study, these effects were investigated using two innovative methods in a large birth cohort. Using maternal‐effects genome complex trait analysis (M‐GCTA), the effects of offspring genotype, maternal or paternal genotypes, and their covariance on offspring internalizing problems were estimated in 3,801 mother–father–child genotyped trios. Next, estimated genetic correlations within pedigree data, including 10,688 children, were used to estimate additive genetic effects, maternal and paternal genetic effects, and a shared family effect using linear mixed effects modeling. There were no significant maternal or paternal genetic effects on offspring anxiety or depressive symptoms at age 8, beyond the effects transmitted via the genetic pathway between parents and children. However, indirect maternal genetic effects explained a small, but nonsignificant, proportion of variance in childhood depressive symptoms in both the M‐GCTA (~4%) and pedigree (~8%) analyses. Our results suggest that parental effects on offspring internalizing problems are predominantly due to transmitted genetic variants, rather than the indirect effect of parental genes via the environment.

## 1 INTRODUCTION

A key issue yet to be resolved in child psychiatry is to what extent associations between parental factors and offspring internalizing problems, such as anxiety and depression, are due to genetic effects, direct environmental effects, or both. Well established risk factors for childhood internalizing problems include exposure to maternal or paternal psychiatric disorders (Côté et al., 2018; Goodman et al., 2011; Ramchandani & Psychogiou, 2009), the parentally provided rearing environment that the child experiences (e.g., parenting style or harsh punishment) (Sangawi, Adams, & Reissland, 2015), and the broader family environment (e.g., marital instability or financial hardship) (Cui, Donnellan, & Conger, 2007; Reiss, 2013). While these associations may be explained by direct environmental effects from parent to child, the relationship is likely to be confounded by shared genetics as each parent passes on 50% of their DNA to their offspring. Moreover, parental environmental effects may still be mediated by the parental genome, acting over and above the transmission of genes from parent to child (Wolf & Wade, 2009). These nontransmitted parental genetic effects may act via the intrauterine environment or the rearing environment that the parent provides for the child. Insight into mechanisms underlying parental influences on offspring internalizing problems is of importance as it could inform both prevention and treatment strategies. Disentangling the effect of transmitted and nontransmitted genetic components, and other environmental sources of variation can only be resolved by genetically informative designs. This study incorporates two novel methodologies to investigate maternal and paternal genetic effects on offspring internalizing problems.

So far, knowledge on genetic and environmental parental influences on offspring internalizing symptoms has largely relied on twin and family based designs rooted in quantitative genetics. Findings from 50 years of twin research estimate that ~40% of the variance within individual differences in childhood internalizing problems is due to genetic factors and up to ~36% is due to the common family environment, which encompasses parental factors that account for similarities within the offspring (Fedko et al., 2017; Polderman et al., 2015; Wesseldijk et al., 2017). The remaining variance is explained by unique environment effects (unshared between twins and siblings), which can also include parental factors. Studies using family‐based designs show evidence of environmental transmission of depressive and anxious symptoms from parent to child, over and above the influence of shared genes (Gjerde et al., 2017; Gjerde et al., 2018; Hannigan, Eilertsen, et al., 2018; McAdams et al., 2015; Rice, Harold, & Thapar, 2005; Silberg, Maes, & Eaves, 2010). In terms of specific parenting behaviors, genetically sensitive designs indicate that over‐reactive parenting (Marceau et al., 2015), harsh parenting (Bridgett et al., 2018), and parental criticism (Horwitz & Neiderhiser, 2011) are associated with more internalizing problems in the offspring, whereas parental expressed affection (McAdams et al., 2017) and a good parent–child relationship quality (Hannigan, Rijsdijk, et al., 2018) are associated with positive offspring self‐worth and fewer internalizing problems respectively. This body of literature highlights that the parentally provided environment is an important contributor to the development of offspring internalizing problems. However, such environmental effects on offspring behavior may have an underlying genetic contribution in the parents (McGuire, 2003), which can be investigated by incorporating information from the parental genome in a parent–offspring design.

In the current genomics era of research, the latest developments in methods of polygenic analyses provide new ways to improve our understanding of the mechanisms underlying parental influence on offspring internalizing problems. Genome‐wide complex trait analysis (GCTA) is used to investigate the impact that variation in measured genetic factors has on behavior (Yang et al., 2010; Yang, Lee, Goddard, & Visscher, 2011). Using genome‐based restricted maximum likelihood (GREML) analyses, common genetic variants are studied to examine the extent to which genetic similarity between unrelated individuals is associated with phenotypic similarity. The additive genetic effect of measured single nucleotide polymorphisms (SNPs) currently explains up to 14% of variance in stable emotional problems during childhood (Cheesman et al., 2018). In samples that, along with data on offspring genotypes and phenotypes, have data available on parental genotypes, a novel extension of the approach used in GCTA can be applied to additionally estimate the contribution of parental genotype to offspring behavior.

Maternal‐effects GCTA (M‐GCTA) (Eaves, Pourcain, Smith, York, & Evans, 2014) uses SNP data to investigate whether variance in an offspring trait can be explained by the effect of the maternal genotype, over and above the transmission of genes from mother to child. In other words, this maternal effect captures the environmental influence of the mother on offspring behavior through genetically influenced maternal traits, for example, through the intrauterine environment or postnatal care. Additionally, M‐GCTA uses the covariance between the direct effect of the offspring genotype and the indirect effect of the maternal genotype to estimate whether genes that contribute to the maternal effect when present in the mother also contribute to the additive genetic effect when present in the offspring. It therefore tests for a passive gene–environment correlation wherein the maternal environment a child is exposed to is correlated with the child's genotype. The M‐GCTA method has not been applied to investigate parental influences on behavioral traits in offspring thus far, but could be a useful technique to capture the impact of parental genetic effects on offspring internalizing behaviors.

Indirect parental genetic effects can also be investigated by a quantitative genetics approach making use of large‐scale family data and extended pedigree information (Merikangas, 2018). Using estimated genetic correlations between known relatives, we examine parental genetic effects on internalizing problems in children and test whether M‐GCTA results replicate. In previous studies, maternal genetic effects on offspring phenotypes were examined by using an extended children‐of‐twins design to estimate the covariance between pairs of individuals with different degrees of relatedness (Magnus, 1984a, 1984b). For instance, it is known that children of monozygotic twins are as genetically similar to their aunt or uncle as they are to their mother or father (McAdams et al., 2014). By comparing the phenotypic covariance between full siblings or children of monozygotic twins (who have 100% of maternal or paternal genetic factors in common) to those whose mothers or fathers are full siblings (share 50% of maternal or paternal genetic factors) or half‐siblings (share 25% of maternal or paternal genetic factors), while taking into account the covariance explained by the other parent and the shared environment for children living in the same family, family data can be used to investigate maternal or paternal genetic effects on offspring behavior.

The aim of this study is to investigate the environmental effect of nontransmitted maternal and paternal genetic factors on offspring internalizing problems. We use data from the Norwegian Mother, Father and Child study (MoBa), a distinctive cohort with extensive data available on over 75,000 complete family trios (mothers, fathers and offspring), including 11,000 genotyped trios. The MoBa dataset provides the unique opportunity to simultaneously study both maternal and paternal influences on offspring behavior. We first use the M‐GCTA method to decompose genetic effects by estimating how variance in offspring internalizing problems is explained by offspring genetic effects, nontransmitted maternal or paternal genetic effects, and a gene–environment correlation between the two. Next, we construct familial genetic correlations using large‐scale pedigree data to clarify the effects of offspring genes, maternal or paternal genetic effects, and shared family effects.

## 2 METHODS

### 2.1 Sample

The Norwegian Mother and Child Cohort Study (MoBa) is a population‐based pregnancy cohort study conducted by the Norwegian Institute of Public Health. Participants were recruited from all over Norway from 1999 to 2008 (Schreuder & Alsaker, 2014). The women consented to participation in 41% of the pregnancies. The cohort now includes 114,500 children, 95,200 mothers, and 75,200 fathers (Magnus et al., 2016). The current study is based on version 10 of the quality‐assured data files released for research in 2018. After birth, information on offspring and maternal outcomes was gathered through maternal‐rated questionnaires at regular follow‐up intervals, currently up to age eight. Parent and infant DNA samples were collected at birth and stored in a biobank (Paltiel et al., 2014). Of these, 11,000 randomly selected trios (mother, father, offspring) were genotyped as part of the HARVEST project (Magnus et al., 2016). We identified 4,645 families with data on internalizing problems available at age 8 and restricted the M‐GCTA analyses to these individuals.

We linked the MoBa dataset to the Medical Birth Registry of Norway (MBRN) to identify siblings among the parents participating in the MoBa study. The MBRN contains a record of all births in Norway from 1967 onward. For same‐sex twin pairs in the parents and offspring generations, zygosity was determined via either genotyping or a twin questionnaire. After exclusion of individuals without any relatives or with missing phenotype data at age eight, the final sample for the pedigree analyses included 10,688 children from 1,552 independent pedigrees (no shared grandparents).

The establishment and data collection in MoBa is based on regulations related to the Norwegian Health Registry Act. The current study was approved by The Regional Committee for Medical Research Ethics (REK 2013/863). Details of all available data are available on the Norwegian Institute of Public Health's website (https://www.fhi.no/en/studies/moba/for-forskere-artikler/questionnaires-from-moba/).

### 2.2 Measures

We investigated two maternally rated internalizing phenotypes at age 8: childhood depression and anxiety symptoms. Childhood depressive symptoms were measured using the parent version of the Short Mood and Feelings Questionnaire (SMFQ) (Angold, Costello, Messer, & Pickles, 1995). The 13‐item scale is based on DSM‐III‐R criteria for depression and consists of descriptive phases regarding how the child had felt or behaved recently. Childhood anxiety symptoms were measured using Birmaher's shortened version of the Screen for Child Anxiety‐Related Disorders (SCARED) consisting of five items (Birmaher et al., 1997). SCARED is a multidimensional questionnaire designed to measure DSM‐defined anxiety symptoms. For both scales, mothers rated how true statements describing their child's recent behaviors were using a 3‐point scale (1 = Not true, 2 = Sometimes true, 3 = True). Based on these measures, childhood depression and anxiety scores were calculated with maximum allowed missingness of two items from the SMFQ and one item from the SCARED questionnaire, per individual. Missing items were imputed with the mean of the nonmissing responses.

### 2.3 Genotyping

MoBa parents and offspring were genotyped using Illumina Human Core Exome Bead chips 12 version 1.1 and 24 version 1.0 and imputed based on the Haplotype Reference Consortium (McCarthy et al., 2016) reference set. Preimputation quality control procedures and imputation processes are described in detail elsewhere (Helgeland et al., 2019). Postimputation, genetic data from the two chips was merged based on overlapping SNPs, according to the procedure used by Fedko et al. (2015). Four and a half million high quality SNPs (imputation info score > 0.9, minor allele frequency > 0.05) were used in downstream analyses.

### 2.4 Statistical analyses

#### 2.4.1 GCTA and extended GCTA analyses

GCTA (Yang et al., 2011) was used to estimate the proportions of variance in depressive and anxiety symptoms that were explained by genome‐wide SNPs in the offspring. First, a genetic relationship matrix (GRM) was calculated to estimate the genetic relationships between pairs of unrelated children based on all autosomal SNPs in the imputed genotype dataset. Cryptic relatedness in the sample was removed using a genetic correlation cut‐off threshold of 0.025. GREML analyses, performed in GCTA, were used to estimate the variance in childhood depression and anxiety symptoms that was explained by the genotyped SNPs (Yang et al., 2010). The analysis adjusted for gender, genotyping batch effects, and the first 10 principal components to account for population stratification.

To resolve nontransmitted maternal and paternal genetic effects, the imputed genotype dataset was split into mother–offspring and father–offspring datasets using Plink 1.96 (Purcell et al., 2007). The M‐GCTA tool (Qiao et al., 2019) was used to construct GRMs indicating genetic similarity between unrelated offspring, unrelated mothers or unrelated fathers, and unrelated mother–offspring or father–offspring pairs. A correlation cut‐off threshold of 0.025 was applied to exclude cryptic relatedness within the groups of mothers, fathers, and offspring. GREML analyses were carried out to examine the extent to which genetic similarity between unrelated parents, as well as unrelated parent–offspring pairs, was associated with similarity in offspring internalizing behaviors. If unrelated parents that were more similar genetically had offspring that were more similar than expected based on the offspring genetic similarity, this would indicate an effect of the nontransmitted parental genotype on offspring internalizing problems. We estimated the proportion of variance in childhood depression and anxiety symptoms that was explained by the offspring's genotype (A), maternal or paternal genotype (M/F), the covariance between the offspring and maternal or paternal genotypes (Q), and the residual environmental component (E). To test for significance, this full model was tested against the classical GCTA AE model. The analysis was performed separately for mother–offspring and father–offspring pairs for childhood depression and anxiety. Gender, genotyping batch, and the first 10 principal components based on the offspring GRM were included as covariates in the analyses.

#### 2.4.2 Pedigree analyses

**FIGURE 1:**
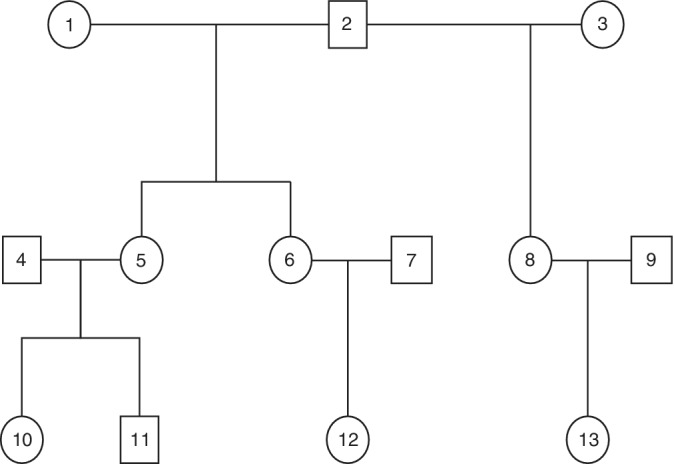
Pedigree figure showing an example of relations between children of siblings included in the pedigree analyses. Individuals 10–13 represent the offspring generation, 4–9 represent their parents, and 1–3 represent their grandparents. Offspring 10 and 11 are full siblings. As Mothers 5 and 6 are full siblings, Offspring 12 is the cousin of Offspring 10 and 11. As Mother 8 is the half‐sibling of Mothers 5 and 6, Offspring 13 is the half‐cousin of Offspring 10, 11, and 12. Offspring 10 and 11 share 50% of additive genetic effects, 100% of maternal effects, 100% of paternal effects, and 100% shared family effects. With Offspring 12, they share 25% of additive genetic effects, 50% of maternal genetic effects, no paternal effects, and no shared family effects. With Offspring 13, Offspring 10, 11, and 12 share 12.5% of additive genetic effects, 25% of maternal genetic effects, no paternal effects, and no shared family effects

Using linkage between MoBa and MBRN, we derived expected genetic correlations among known relations of children in the sample (e.g., Figure 1). To capture offspring additive genetic effects, we made use of monozygotic and dizygotic twin correlations, as well as correlations between siblings, half‐siblings, cousins, and half‐cousins (children of half‐siblings). Maternal effects were examined by comparing correlations between children of the same mother and children whose mothers were monozygotic twins (these children share 100% of maternal genetic effects) to children whose mothers were full siblings (share 50% of maternal genetic effect) and children whose mothers were half‐siblings (share 25% of maternal genetic effect). If children who shared the same mother, or whose mothers were monozygotic twins, were more alike than children whose mothers were full siblings or half‐siblings, this would indicate a maternal genetic effect on offspring internalizing problems. To account for influences due to the other parent and the shared family environment, we further tested for a shared family effect, which was shared among children of the same mother and father. In a separate model, paternal effects were examined using the same structure, but focusing on fathers of children instead of mothers. The number of different correlations within each type of effect are tabulated in Table 1.

**TABLE 1:** Number of distinct correlations between pairs of children for each of the included random effects

Type of effect	1/16	1/8	1/4	1/2	1
Additive genetic	95	2,339	101	4,235	116
Maternal genetic	0	0	57	1,154	4,411
Paternal genetic	0	0	30	857	4,382
Shared environment	—	—	—	—	4,351

*Note:* Additive genetic effect: “1” = monozygotic twins, “2” = dizygotic twins or full siblings, “1/4” = half‐siblings, “1/8” = cousins, “1/16” = half‐cousins. Maternal or paternal genetic effect: “1” = full siblings or children of monozygotic twins, “1/2” = children of full siblings, “1/4” = children of half‐siblings. Shared family effect: “1” = children with the same mother and father (full siblings).

We modeled the covariance structure among the childhood phenotypes, depression and anxiety symptoms, as arising from offspring additive genetic effects (A), indirect maternal and paternal genetic effects (M/F), shared family effects (C), and environmental effects unique to the individual (E). While individuals could be correlated with each other within each type of effect, the different types of effects were assumed to be independent of each other, that is, no gene–environment correlation. Variance components associated with the different types of random effects were estimated using a linear mixed effects model (Pawitan, Reilly, Nilsson, Cnattingius, & Lichtenstein, 2004) in software package R, version 3.4.4. In all analyses, gender of offspring was included as a covariate.

## 3 RESULTS

After quality control procedures, the extended GCTA analyses included data on up to 3,801 trios, while data on 10,688 children were included in the pedigree analyses. Sample descriptive statistics are shown in the Supplementary Information.

### 3.1 GCTA and extended GCTA analyses

We present the results of the GCTA analyses in Table 2. In the standard GCTA models, offspring additive genetic effects from measured SNPs explained close‐to‐significant variance in childhood depressive symptoms (0.10, 95% confidence intervals [CI]: −0.3 to 0.23) and significant variance in childhood anxiety symptoms (0.17, 95% CI: 0.03–0.31). The extended GCTA models including the parental effects did not show a better fit than the standard AE model. The CI showed that none of the variance components were significant, although maternal and paternal genotypes explained small proportions of variance in childhood depressive symptoms (0.04, 95% CI: −0.17 to 0.26 and 0.06, 95% CI: −0.16 to 0.28, respectively).

**TABLE 2:** Results from GCTA and extended GCTA analyses

	*A* (*SE*)	M/F (*SE*)	*Q* (*SE*)	*G* (*SE*)	*E*	df	*p*	*N*
Depressive symptoms (SMFQ)								
Standard GCTA	0.10 (0.07)	—	—	0.10 (0.07)	0.90	1	.053	3,794
Maternal effects GCTA	0.14 (0.11)	0.04 (0.11)	0.00 (0.09)	0.18 (0.12)	0.82	2	.4	3,030
Paternal effects GCTA	0.11 (0.11)	0.06 (0.11)	0.00 (0.08)	0.17 (0.12)	0.83	2	.4	3,059
Anxiety symptoms (SCARED)								
GCTA	0.17 (0.07)	—	—	0.17 (0.07)	0.83	1	.007	3,801
Maternal effects GCTA	0.16 (0.11)	0.00 (0.10)	0.00 (0.08)	0.16 (0.12)	0.84	2	.5	3,038
Paternal effects GCTA	0.03 (0.11)	0.00 (0.11)	0.06 (0.09)	0.09 (0.12)	0.91	2	.3	3,067

*Note:* Model parameters are: *A* variance due to direct additive genetic (“offspring” effects), *M* variance due to indirect maternal genetic effects on offspring phenotype (“maternal effects”), *F* variance due to indirect paternal genetic effects on offspring phenotype (“paternal effects”), *Q* phenotypic variance due to covariance of direct and indirect genetic effects, *G* variance due to combined direct and indirect genetic effects and the residual *E* (“unique environmental effects”). *SE*: standard error, *p* = *p* value, *N* = sample size. The *p*‐value is calculated by comparing the full model to the model with the offspring component only.

Abbreviations: SCARED, Screen for Child Anxiety Related Disorders; SMFQ, Short Mood and Feelings Questionnaire.

### 3.2 Pedigree analyses

Table 3 shows correlations in anxiety and depressive scores between related individuals. There were no shared family effects on offspring depression or anxiety symptoms; therefore, the shared family effect was omitted from both models (Table 4). Offspring additive genetic effects were present for both depression and anxiety symptoms, as model fitting showed that omitting the offspring genetic effect significantly worsened model fits (depressive symptoms: *χ* ^2^ = 338.38, *p* < 2e‐16, anxiety symptoms: *χ* ^2^ = 166, *p* < 2e‐16). The maternal effect explained a small percentage of variance in offspring depressive symptoms (7.6%), but this was not significant as the model including the maternal effect was no different to the model which only included offspring genetic effects (*χ* ^2^ = 1.71, *p* = .19). There was no paternal effect on offspring depressive symptoms, and no maternal or paternal effects on offspring anxiety symptoms.

**TABLE 3:** Phenotypic correlations between children that were present in the pedigree analyses

	Depression symptoms (95% CI)	Anxiety symptoms (95% CI)	*N*
Monozygotic twins	0.553 (0.412–0.668)	0.674 (0.560–0.763)	116
Dizygotic twins	0.162 (0.046–0.273)	0.211 (0.097–0.320)	282
Full siblings	0.272 (0.242–0.302)	0.152 (0.120–0.183)	3,702
Half‐siblings	−0.029 (−0.333–0.281)	0.345 (0.041–0.590)	45
Cousins	0.053 (−0.012–0.117)	0.018 (−0.047–0.082)	917
Half‐cousins	0.283 (0.016–0.512)	−0.005 (−0.272–0.263)	54

*Note: N* = number of pairs used to calculate each correlation. 95% CI = 95% confidence intervals. Pairwise correlations presented are indicative, but not representative of all data within the analyses. Correlations were calculated by using at most one pair from a nuclear family and with each individual only able to partake in one pairing per correlation. Thus, children with more than one sibling, half‐sibling, cousin, or half‐cousin are underrepresented in this table but are included in the linear mixed effects model.

**TABLE 4:** Results from the pedigree analyses

Phenotype	Model	A (*SE*)	M/F (*SE*)	C (*SE*)	E (*SE*)
Depression symptoms	Maternal effects	0.419 (0.12)	0.076 (0.06)	0.000 (0)	0.505 (0.06)
	Paternal effects	0.554 (0.11)	0.000 (0)	0.006 (.06)	0.440 (0.05)
Anxiety Symptoms	Maternal effects	0.377 (0.03)	0.000 (0)	0.000 (0)	0.623 (0.03)
	Paternal effects	0.377 (0.03)	0.000 (0)	0.000 (0)	0.623 (0.03)

*Note:* SE, standard error. Model parameters are: *A* variance due to direct additive genetic (“offspring” effects), *M* variance due to maternal environmental effect (“maternal effects”), *F* variance due to paternal environmental effect (“paternal effects”), *C* variance due to the shared family effect and the residual *E* (“unique environmental effects”).

## 4 DISCUSSION

We set out to resolve the impact of nontransmitted parental genetic factors on offspring internalizing problems during childhood using two complementary approaches: M‐GCTA analyses and pedigree analyses. The extended GCTA analyses used molecular data from genotyped trios to estimate the contribution of maternal and paternal genetic effects on offspring internalizing problems, beyond the effects of transmitted genes from parents to offspring, and further investigated whether there was evidence of a passive gene–environment correlation. The pedigree analyses investigated maternal and paternal genetic effects using estimated genetic correlations from rich family data, and additionally examined whether there was a shared family effect in full siblings. In both the M‐GCTA and pedigree analyses, there were no significant nontransmitted maternal or paternal genetic effects on childhood depression or anxiety symptoms. The M‐GCTA analyses showed no evidence of a passive gene–environment correlation for childhood depression or anxiety symptoms, and the pedigree analyses found no shared family effect.

Focusing on the results for offspring depressive symptoms, findings from the M‐GCTA and pedigree analyses converged to show that a small proportion of variance (between 4 and 8%) was explained by nontransmitted maternal genetic effects, although the estimate was not significant in either of the analyses. The contribution of these maternal genetic effects led to an increased proportion of variance explained in the extended GCTA (18%), compared to when maternal genetic effects were not included in the analyses (10%). While the large confidence intervals signify insufficient power, the consistency of the estimate using two independent methodologies suggests that the true contribution of maternal genetic effects on offspring depressive symptoms is likely not far from this estimate. Therefore, we predict that although a larger sample size would be required to find a significant maternal genetic effect on symptoms of depression, the size of this effect is likely to remain relatively small. Previous family based studies have found small (0.05) (Rice et al., 2005) to moderate (0.28) (McAdams et al., 2015) direct environmental effects of concurrent maternal depression, but no effect of prenatal depressive symptoms (Hannigan, Eilertsen, et al., 2018), on offspring internalizing problems after taking into account confounding due to shared mother–offspring genes. Bearing these results in mind, the findings of the current study suggest that maternal genetic factors may account for a small proportion of the overall environmental effects on offspring behavior that arise due to the mother. With regard to paternal genetic effects on offspring depressive symptoms, results from the two methodologies were discrepant. A small effect was observed in the M‐GCTA analyses (explaining 6% of the variance), but was not replicated in the pedigree analyses. As paternal effects are rarely studied, in part due to limited availability of paternal data, more research is required to interpret this inconsistent finding and elucidate the impact of paternal genome on offspring depression symptoms.

Results from the M‐GCTA and pedigree analyses converged again when looking at nontransmitted parental genetic effects on offspring anxiety symptoms. There were no effects of maternal or paternal genotype on anxiety symptoms, using either of the methodologies. There are two possible explanations for this; there may have been insufficient power to detect indirect parental genetic effects on anxiety symptoms, or childhood symptoms of anxiety may be unaffected by indirect parental genetic effects. Further research is required to clarify which of these is the case. However, if the latter is true it may hold implications for research on parental influences on internalizing problems that group anxious and depressive symptoms together, as there may be different effects underlying the parent–offspring associations. Indeed, it has previously been suggested that while genetic influences underlying anxiety and depression are not disorder‐specific, environmental effects could be specific and unshared across the two disorders (Kendler, Heath, Martin, & Eaves, 1987). Therefore, it may be that genetically influenced parental characteristics have some influence on offspring depressive symptoms, but not anxiety symptoms. This requires further investigation, as although the current findings suggest a small indirect maternal genetic effect on offspring depressive symptoms, the results were not statistically significant.

Previous research found gene–environment correlation effects on internalizing problems in childhood (Hannigan, Rijsdijk, et al., 2018; Narusyte et al., 2008). However, as the parental effects were nonsignificant in the current study, it was impossible to detect such an effect, even if it were present. More power in the M‐GCTA analyses would be needed to detect whether gene–environment interplay underlying offspring internalizing problems arises due to the indirect effect of the parental genome. Alternatively, it is also possible that the gene–environment correlations observed in offspring internalizing problems within existing research do not act via parental factors that are genetically influenced. The current study also did not find a shared family effect (reflecting the influence of the other parent and the shared family environment) on depression or anxiety symptoms, within the pedigree‐based analyses. Within previous research, estimates of variance explained by the common family environment are broad and range from 0 to 0.32 (Fedko et al., 2017; Polderman et al., 2015; Wesseldijk et al., 2017). The ability to detect the effect varies, depending on the population and sample size. Finally, the pedigree analyses in the current study showed that large amounts of variance in depressive and anxious symptoms were explained by unique environmental effects. It is important to note that these may include the effects of parental behaviors toward the child that are not genetically influenced, and are child specific.

In the context of broader literature, estimates of the contribution of additive genetic effects to variance in depression (45%) and anxiety (38%) from the pedigree analyses were in line with existing findings which estimate that ~40% of the variance in internalizing problems in childhood is due to genetic factors (Polderman et al., 2015). Our results confirm that individual differences in childhood anxiety and depression in childhood have a substantial underlying genetic component. In molecular research, the maximum estimate of SNP heritability of internalizing problems from previous research is 14% (Cheesman et al., 2018). The estimates from the current study are close to this, with measured genetic variants explaining 10% of the variance in depressive symptoms (not significant) and 17% of the variance in anxiety symptoms (significant). The gap in heritability estimates based on the pedigree analyses versus GCTA analyses is not unexpected, and is widely recognized in existing literature (Cheesman et al., 2017; Maher, 2008; Manolio et al., 2009).

The current study has a number of strengths. We used methodological triangulation in investigating our research question to determine whether results from quantitative and molecular genetics approaches converged. To our knowledge, this is the first application of the M‐GCTA technique to examine parental genetic effects on mental health outcomes, as the method has previously only been applied to study physical characteristics such as birth length and weight (Eaves et al., 2014; Horikoshi et al., 2016; Qiao et al., 2019). Furthermore, much of the research investigating parental contribution to offspring internalizing problems in childhood has primarily focused on mothers (Sawyer, Zunszain, Dazzan, & Pariante, 2018), even though paternal factors also exert an influence on offspring behavior. This study pays equal attention to the contribution of maternal and paternal influences. The study design is resourceful as it does not require direct measurement of parental phenotypes in order to study parental influences on offspring internalizing problems. This is an advantageous approach for cohorts that do not have measurements of parental behaviors, to still answer research questions investigating parental effects on offspring behavior. The approach is also useful when the mechanisms through which parents have an effect are unclear and the relevant variables cannot be easily identified.

The results of this study should be considered in the context of certain limitations. First, the M‐GCTA analyses were underpowered to detect maternal or paternal genetic effects on offspring internalizing problems. Despite a large sample of genotyped trios available (11,000), after quality control procedures and exclusion of missing data, the sample was limited to between 3,000 and 3,800 pairs per analysis. This yielded limited power (0.57) to detect a maternal or paternal genetic effect of 0.05, in proportion of variance explained. It is now estimated that at least 10,000 pairs are required to detect maternal or paternal genetic effects (Moen, Hemani, Warrington, & Evans, 2019). Second, in cohort studies with long‐term follow‐up such as MoBa, biases in study participation can impact the results. It has already been shown that participation at baseline was related to maternal education (Biele et al., 2019). Furthermore, there was substantial study dropout as only 47% of the original sample had data available at age 8 (Schreuder & Alsaker, 2014). If families of children with internalizing problems withdrew from the study or were less likely to participate, this would reduce coverage of the higher end of the distribution within the sample. This could be important if severe cases have different underlying mechanisms. In investigating this, we found that children whose mothers answered questions on internalizing behaviors at two measurement points (age 3 and 8) showed fewer internalizing symptoms on average, than those who responded at one time point, either age 3 or age 8 (Supplementary Information). Based on this selective nonresponse bias, the current findings may not extend to individuals with more severe internalizing problems, if they are differentially impacted by indirect parental genetic effects. Finally, although the use of maternal ratings to define offspring internalizing behaviors is beneficial as mothers are considered good informants on early life behaviors among children (Loeber, Green, & Lahey, 1990), it could also be a potential limitation. In using maternal ratings of offspring behavior to identify maternal effects, we are restricted in our ability to distinguish real environmental effects from rater bias effects. Sources of rater bias are stereotyping, employing different normative standards, or having certain response styles (e.g., judging problem behaviors more or less severely). Previous twin research shows that 10–20% of the variance in internalizing behaviors is accounted for by rater bias (Bartels, Boomsma, Hudziak, van Beijsterveldt, & van den Oord, 2007; Fedko et al., 2017; Wesseldijk et al., 2017). A large study with behavioral observations would be an opportunity to overcome these effects of rater bias, although these observations might also be biased and are not feasible in large population‐based cohorts.

There are several additional avenues for future investigations in light of the current findings. We first note that larger sample sizes are needed to generate enough power to adequately estimate internalizing problems variance components based on SNP effects. To achieve this, it would be beneficial to combine data from multiple cohorts in order to maximize the number of genotyped individuals available. In cohorts with large amounts of family data available, the influence of other family members, such as siblings or adoptive parents, could additionally be studied using the M‐GCTA technique. The method would also very well compliment other recently developed genetic nurture methodologies, such as exploring the effect on nontransmitted parental alleles on offspring behavior (Kong et al., 2018). Finally, the current study specifically focuses on nontransmitted maternal and paternal genetic effects on offspring internalizing problems. Future research may wish to focus on other mechanisms that account for the influence of parental factors on offspring internalizing problems. For instance, in animal models mother–offspring interactions have been shown to influence DNA methylation in the offspring, leading to changes in gene expression, that may be related to offspring behavior (Jensen Peña & Champagne, 2012; Kappeler & Meaney, 2010).

In summary, we applied two distinct methodologies to investigate maternal and paternal genetic effects on offspring internalizing problems during childhood. Variation in offspring internalizing problems was predominantly due to offspring additive genetic effects rather than indirect maternal or paternal genetic sources of variation. However, the pattern of results suggests that indirect maternal genetic effects may account for a small proportion of variation in offspring depressive symptoms in childhood.

## CONFLICT OF INTEREST

The authors declare no potential conflict of interest.

## AUTHOR CONTRIBUTIONS

Eshim S. Jami analyzed the genotype data and Espen Moen Eilertsen analyzed the pedigree data. Eshim S. Jami, Anke R. Hammerschlag, Meike Bartels, and Christel M. Middeldorp wrote the manuscript. The project was supervised by Anke R. Hammerschlag, Eivind Ystrøm, Meike Bartels, and Christel M. Middeldorp. Zhen Qiao and David M. Evans provided support for the analysis and interpretation of the genotype data. All authors critically revised the manuscript and approved the final version.

## Supporting information


**Appendix** **S1:** Supplementary Information
