# Supplementary material for: Maternal and paternal effects on offspring internalizing problems: Results from genetic and family‐based analyses
Source: Am J Med Genet B Neuropsychiatr Genet. 2020 May 1;183(5):258–67. doi: 10.1002/ajmg.b.32784 (PMC7317352; doi:10.1002/ajmg.b.32784)
Supplement: Supplementary file 1 — Appendix S1: Supplementary Information [file AJMG-183-258-s001.docx]

Maternal and paternal effects on offspring internalising problems: results from genetic and family-based analyses

Jami *et al.*

**Supplementary Information**

**Sample description**

Sample and phenotype descriptive statistics are shown in Table 1.

**Table 1** Sample and phenotype descriptive statistics

|  | Depressive symptoms  (SMFQ) | Anxiety symptoms  (SCARED) |
| --- | --- | --- |
| Percentage male | 52.3% | 52.4% |
| Age mean | 8.129 | 8.129 |
| Age standard deviation | 0.12 | 0.12 |
| Age range | 7.67 – 9.0 | 7.67 – 9.0 |
| Scale mean | 14.74 | 5.975 |
| Scale variance | 5.69 | 1.39 |
| Scale range | 13 – 35 | 4 – 15 |
| Scale Cronbach’s alpha | 0.81 | 0.55 |

**Sex differences in anxiety and depression symptom scores**

Sex differences were observed in anxiety symptom scores from the SCARED questionnaire. Girls scored significantly higher than boys (mean girls: 6.06, mean boys: 5.91, *t* = -4.4, *p* <.001). There were no sex differences in depressive symptom scores (mean girls: 14.73, mean boys: 14.73, t = -0.008, *p* = 0.99).

**Correlation between depression and anxiety scores**

The correlation between summary scores for depressive and anxiety symptoms was low (0.22).

**Biases in survey participation**

The study data was drawn from version 10 of the quality-assured MoBa data files, which includes ~58,000 individuals for whom scores for internalising symptoms at age 3 were available, and ~43,000 individuals with data on anxiety and depressive symptoms at age 8 (Table 2).

**Table 2** Sample sizes of overall measurements of internalising symptoms available within MoBa

| Measurement(s) available | Number of individuals |
| --- | --- |
| INT age 3 | 58,348 |
| ANX age 8 | 43127 |
| DEP age 8 | 43006 |
| INT age 3 & ANX age 8 | 35,670 |
| INT age 3, but no ANX age 8 | 22,678 |
| ANX age 8, but no INT age 3 | 7,457 |
| INT age 3 & DEP age 8 | 35,571 |
| INT age 3, but no DEP age 8 | 22,777 |
| DEP age 8, but no INT age 3 | 7,435 |

INT = internalising problems, measured using the Child Behaviour Checklist (CBCL); ANX = anxiety symptoms, measured using the Screen for Child Anxiety Related Disorders (SCARED); DEP = depressive symptoms, measured using the Short Mood and Feelings Questionnaire (SMFQ)

Individuals for whom survey data at both age 3 and age 8 were available had, on average, fewer earlier internalising problems at age 3, compared to individuals who only had data available at age 3 (Table 3). Additionally, individuals with measurements at both time points (age 3 and age 8) also showed fewer symptoms of anxiety or depression at age 8, than individuals who participated at age 8, but had no data available at age 3. Together, these analyses show that children whose mothers answered questions on internalising behaviours at two measurement points (age three and eight) showed fewer internalising symptoms on average, than those who responded at one time point, pointing to a selective non-response bias.

**Table 3** Results of t-tests investigating mean difference between symptom scores, depending on availability of measurements

| Group 1 | Group 2 | Comparison measure | Mean Group 1 (SD) | | Mean Group 2 (SD) | 95% CI for estimated mean difference | p-value |
| --- | --- | --- | --- | --- | --- | --- | --- |
| INT age 3 & ANX age 8 | INT age 3, but no ANX age 8 | INT scores age 3 | 11.17 (1.95) | 11.29 (2.05) | | -0.16, -0.09 | <.001 |
| INT age 3 & DEP age 8 | INT age 3, but no DEP age 8 | INT scores age 3 | 11.16 (1.95) | 11.29 (2.04) | | -0.16, -0.09 | <.001 |
| ANX age 8 & INT age 3 | ANX age 8, but no INT age 3 | ANX scores age 8 | 6.02 (1.18) | 6.09 (1.26) | | -0.10, -0.04 | <.001 |
| DEP age 8 & INT age 3 | DEP age 8, but no INT age 3 | DEP scores age 8 | 14.85 (2.42) | 14.94 (2.59) | | -0.15,-0.03 | 0.003 |

CI = confidence intervals; INT = internalising problems, measured using the Child Behaviour Checklist (CBCL); ANX = anxiety symptoms, measured using the Screen for Child Anxiety Related Disorders (SCARED); DEP = depressive symptoms, measured using the Short Mood and Feelings Questionnaire (SMFQ)
